# Detoxification of Implant Surfaces Affected by Peri-Implant Disease: An Overview of Surgical Methods

**DOI:** 10.1155/2013/740680

**Published:** 2013-08-04

**Authors:** Pilar Valderrama, Thomas G. Wilson Jr

**Affiliations:** ^1^Department of Periodontics, Texas A & M University Baylor College of Dentistry, 3302 Gaston Avenue, Dallas, TX 75246, USA; ^2^Private Practice of Periodontics, 5465 Blair Road, Suite 200, Dallas TX 75231, USA

## Abstract

*Purpose*. Peri-implantitis is one of the major causes of implant failure. The detoxification of the implant surface is necessary to obtain reosseointegration. The aim of this review was to summarize in vitro and in vivo studies as well as clinical trials that have evaluated surgical approaches for detoxification of the implant body surfaces. *Materials and Methods*. A literature search was conducted using MEDLINE (PubMed) from 1966 to 2013. The outcome variables were the ability of the therapeutic method to eliminate the biofilm and endotoxins from the implant surface, the changes in clinical parameters, radiographic bone fill, and histological reosseointegration. *Results*. From 574 articles found, 76 were analyzed. The findings, advantages, and disadvantages of using mechanical, chemical methods and lasers are discussed. *Conclusions*. Complete elimination of the biofilms is difficult to achieve. All therapies induce changes of the chemical and physical properties of the implant surface. Partial reosseointegration after detoxification has been reported in animals. Combination protocols for surgical treatment of peri-implantitis in humans have shown some positive clinical and radiographic results, but long-term evaluation to evaluate the validity and reliability of the techniques is needed.

## 1. Introduction

The vast majority of implants are successful over the long term. However, failure does occur. These failures occur for a variety of reasons. Currently available literature indicates that peri-implant infections are one of the major causes of implant failure. These infections have been related to biofilms colonization of the implant surface that induces an inflammatory response [1]. These conditions are divided into those affecting only the soft tissues (peri-implant mucositis) or those resulting in loss of supporting bone (peri-implantitis) [2]. Infections affecting only the soft tissues can normally be resolved by debriding the area along with increased attention to personal oral hygiene. The clinician's current dilemma is how to optimally deal with infected implant surfaces where partial loss of bone has occurred. To date there have been no human studies demonstrating on histologic level the reattachment of bone to infected implant surfaces. The current belief is that this is a result of the bacteria and their byproducts left on the implant surfaces. As a result, many approaches have been suggested to detoxify these surfaces. This paper will present an overview of the surgical approaches suggested to date. 

Lack of a specific clinical and radiographic definition of peri-implantitis makes it difficult to determine the exact prevalence of the disease. Estimates have ranged from 5% to greater than 60%. The important concept is that this disease is prevalent and the number of implants affected increases the longer these implants have been in place [3]. In a recently published systematic review in which 1497 patients and 6283 implants were followed for more than 5 years, peri-implant mucositis was found in 63.4% patients and around 30.7% of the implants and for peri-implantitis 18.8% and 9.6% respectively, with smokers being at a higher risk for these conditions [4].

Current evidence suggests that peri-implantitis does not respond to traditional nonsurgical therapy [5]. In addition, surgical therapy has been demonstrated to result in significantly reduced probing depth and gains in clinical attachment levels around affected implants [6]. The aim of this review was to summarize the findings of studies that have evaluated therapies for detoxification of the implant body surfaces in studies in vitro and in vivo or in clinical trials evaluating the surgical treatment of peri-implantitis. 

## 2. Materials and Methods

A literature search was performed using MEDLINE (PubMed) from January 1, 1966 to February 20, 2013. The search strategy included the following terms: peri-implantitis treatment and implant surface decontamination. Articles in English were included and the search resulted in 574 articles. Titles and abstracts were screened and the full text of 76 publications reporting on the evaluation of mechanical, and chemical methods as well as lasers used for treatment of contaminated implant surfaces were selected. The articles were evaluated based on the following inclusion criteria: systematic reviews, longitudinal studies reporting on surgical treatment of peri-implantitis, case series, and in vitro studies and in vivo studies reporting histological findings after surface decontamination. The bibliographies of systematic reviews were hand searched. Clinical studies reporting on the nonsurgical treatment of peri-implantitis were excluded. The outcome variables were the ability of the therapeutic method to eliminate the biofilm and endotoxins from the implant surface, the changes in clinical parameters like probing depth, clinical attachment levels, and bleeding on probing; radiographic bone fill and histological reosseointegration.

## 3. Results

Commonly used methods for implant surface detoxification. 

### 3.1. Mechanical Methods

#### 3.1.1. Implantoplasty

When a titanium implant surface has been exposed to the oral cavity and contaminated with bacteria, implantoplasty to completely flatten/smooth the exposed part of the implant using rotary instruments may be indicated [7]. Initially recommended by Lang et al. [8] and reported by Suh et al. [9], this technique aims to reduce the roughness of the titanium surface to decrease plaque adherence since it has been demonstrated that rough surfaces accumulate more plaque than smooth or moderately rough surfaces [10–12]. In vitro studies have shown that the use of diamond polishing devices can remove the coating of the implant surface entirely thus exposing the body of the fixture [13]. There is no consensus about the type of bur to use for implantoplasty. An in vivo study showed that diamond grit and carborundum polishing or just the carborundum give similarly polished surfaces [14].

In a study comparing resective surgery plus implantoplasty with resective surgery alone for the treatment of TPS surfaced implants, a 3-year follow-up in humans with peri-implantitis demonstrated that implantoplasty improves the survival rate (100% versus 77.6%) and prevented further significant marginal bone loss [15]. This approach significantly improved probing depths (PD), clinical attachment levels (CAL), and bleeding (BOP) compared to resective surgery. However the marginal recession was increased in the implantoplasty group [16]. In this study, the authors also used a 25% metronidazole gel and 50 mg/mL solution of tetracycline HCl for decontamination of the implant surface after debridement of the bone defect. Implantoplasty is usually done in combination with antimicrobial therapy. The use of metronidazole and amoxicillin has shown the best results in studies in animals [17]. Implantoplasty has also been combined with regenerative surgery and subepithelial connective tissue graft (SCTG). Schwarz et al., recently published a 6-month follow-up of 10 cases treated with implantoplasty, surface decontamination with saline soaked cotton pellets and xenograft plus collagen membrane, and SCTG and showed a significant reduction in PD, CAL, and soft tissue recession [18].

Implantoplasty followed by further implant surface decontamination with plastic curettes plus saline soaked cotton pellets before bone grafting and membrane placement has shown to significantly improve the clinical parameters like BOP, PD reduction and CAL as well as radiographic bone fill [19, 20]. 

The remnants of the coating of the implant are expected to remain as metal debris in the surrounding tissues [13]. These particles may or may not be associated with clinical adverse events; however this remains to be determined. It is unknown if the treated titanium surface will form titanium oxides that will allow re-osseointegration. An in vitro study has shown, that under proper cooling conditions, implantoplasty does not generate excess temperature increases that can damage soft tissue or bone surrounding the treated implant [21]. One of the major disadvantages of this technique is the increased postoperative recession of the marginal tissues and exposure of the abutment and implant surface which negatively affects the esthetics and increases food impaction. In most situations reattachment of bone to previously toxic implant surfaces is the desirable outcome. Therefore, smoothing of the exposed implant surface as monotherapy is not the optimal approach in many clinical situations.

#### 3.1.2. Air Powder Abrasive: AP

Air powder abrasive (AP) features the use of an abrasive powder, generally sodium bicarbonate, and sodium hydrocarbonate [13], or amino acid glycine [22], propelled by a stream of compressed air to remove biofilm or extrinsic stains from teeth [23]. This instrument applies a mix of water, air, and powder at pressures of 65 to 100 pounds per square inch (psi) [24] and has been demonstrated in in vitro and in vivo studies to be effective in cleaning the previously contaminated implant surfaces [25]. Tastepe et al. (2012) published a review of 27 articles that dealt with the efficacy of this approach in cleaning the implant surface as well as the clinical response to implants treated using this method. The articles analyzed included 19 in vitro studies, 3 in vivo studies, and 4 human studies. They concluded that the cleaning efficiency evaluated by the removal of bacterial endotoxin ranged from 84% to 98% and the removal of the bacteria biofilm was up to 100% in in vitro studies [23]. This approach has not been shown to alter the physical structure of some implant surfaces [26, 27]. However it has been shown that particles of the powder can stay attached to the implant surface after cleaning [27]. In addition, when this approach is used on machined surfaced implants, alterations of the surface topography can occur and large amounts of powder particles attaching to the implant surface have been seen in in vitro studies [28, 29]. How this affects re-osseointegration remains unknown. Tastepe et al. also reported that there was no significant effect on cell response measured as cell attachment and proliferation when compared with control groups [23]. There are no in vivo or human studies in which complete re-osseointegration has been demonstrated by the solo use of air powder abrasive; however, some animal studies have shown bone regeneration with this method when it is combined with bone grafts and membranes for guided bone regeneration [30–32]. Froum et al. proposed a surgical protocol for detoxification of implant surfaces in humans that included AP. They reported significant bone fill and general clinical improvement up to 7.5 years after the use of AP 60 seconds followed by a solution of tetracycline application, followed by a second application of AP for 60 seconds and finally rinsing with 0.12% chlorhexidine for 30 seconds before bone grafting in 38 patients with 51 implants affected by peri-implantitis [33].

It can be concluded that air powder abrasive can contribute to the detoxification of the implant surface and can improve the clinical outcomes when used in combination with surgical regenerative procedures. However, adverse effects like subcutaneous emphysema have been reported with the use of air abrasive around teeth [24] and around implants [34]. While this complication might not occur if the tip if the instrument is cautiously used at a 45° angle to the implant [13], this approach could not be routinely recommended based on the available literature.

#### 3.1.3. Ultrasonic Scaler with a Metal Tip

One of the main concerns of clinicians when trying to clean the implant surface is not knowing the effect of the instrument on the implant body surface. When applied to rough surfaces, this technique has shown in vitro to produce a smoother surface with reduced irregularities and to remove bacteria more efficiently than ultrasonic scalers covered with a plastic tip [35]. The influence that this change can have on the re-osseointegration process or survival rates is unknown. It is also important to consider which therapy can enhance the ease of maintenance of implants. Rough implant surfaces are more susceptible to faster bone loss, once there is peri-implantitis [36]. Therefore, metal tips which have shown to smooth the roughened surface may ease the removal of bacteria using personal oral hygiene [35]. If machined surfaces are altered, the scratches of the surfaces do not significantly affect the amount of plaque that adheres. In fact one in vivo study showed that reduction of the surface roughness, below a certain threshold *R(a)* (0.2 microns), has no major impact on the supra- and subgingival microbial composition [37].

#### 3.1.4. Metal Curettes

An in vitro study using a surface profilometer showed that metal curettes reduce the roughness of rough surfaced implants and decrease the attachment of *Streptococcus sanguini* which is an important early colonizer in the oral cavity [29]. Metallic curettes after 20 seconds of use can remove superficial material from the rough surface of on average 0.83 *μ*m compared to 0.19 *μ*m removed by titanium curettes and ultrasonic tips covered with plastic inserts [26].

#### 3.1.5. Nonmetal Curette Scalers

These instruments can be made of plastic, carbon, resin-reinforced, and resin-un-reinforced.

In vitro studies have shown incomplete removal of biofilm [13]. A systematic review showed that when used to treat smooth surface implants the resulting surfaces were similar to the untreated control; when used to clean rough surfaced implants some particles of the curette material were deposited on the implant surface [7]. In terms of re-osseointegration, in dogs, plastic curettes have shown poor results in terms of re-osseointegration even when used in combination with metronidazole gel [38].

### 3.2. Chemical Methods

#### 3.2.1. Citric Acid (CA)

Citric acid has been widely reported in the literature for the detoxification of the implant surface. There are numerous papers evaluating the in vitro and in vivo effectiveness of this chemical. However, in the articles reviewed here there was no agreement about the concentration and duration of application. In vitro, burnishing CA pH1 with cotton pellet for 1 minute has been shown to significantly decrease the amount of E. coli LPS (LPS count 68 min/mm^2^) on titanium alloy grit blasted surfaces compared to untreated controls (LPS count 197 min/mm^2^). When hydroxyapatite (HA) coated strips were evaluated following CA application the reduction was more profound. This may be explained by the demineralizing effect of CA on HA [39]. In an vitro study, citric acid soaked cotton pellets were rubbed on titanium cylindrical units contaminated with *Porphyromonas gingivalis *endotoxin for one minute and for 2 minutes. The one-minute treatment leads to a reduction of the endotoxin by 85.8% for machined surface, 27% for titanium plasma sprayed, and 86.8% for hydroxyapatite coated titanium implants. The two-minute treatment produced a reduction of 90%, 36.4%, and 92.1%, respectively [40]. From these studies we can conclude that CA is able to significantly decrease the amount of LPS present on machined surface and HA coated implants, but it is not as effective on titanium plasma sprayed implants. As a counterpoint, when CA was tested for the ability to eliminate human biofilms in vivo, CA was unable to inactivate the attached bacterial cells from smooth titanium discs after being submerged on a 40% CA solution for 1 minute [41].

When analyzing the changes induced by CA on the composition of the implant, an in vitro study on retrieved failed contaminated smooth surface implants showed that after a saturated solution of CA was applied for 30 seconds and then rinsed with sterile water the amount of carbon, oxygen, and nitrogen exceeded the amount of titanium on the surface. These contaminants could inhibit re-osseointegration when present [28]. However, in another in vitro study, when a supersaturated solution of CA was used in combination with hydrogen peroxide (HP) and a CO_2_ laser, the viable bacteria were reduced significantly and the final surface composition was comparable to the noncontaminated implant surface with increased levels of titanium and oxygen and decreased amounts of carbon [42]. In re-osseointegration studies in dogs the application of CA for 30 seconds followed by rinsing with saline on dental implants with machined surfaces [43] or Ti-Unite surfaces was evaluated [44]. This was compared to tooth brush and saline for 1 minute and swabbing with HP 10% for 1 minute. All animals were medicated with 150 mg of clindamycin bid for one week. The results showed that CA was as effective as brushing with saline and HP to decontaminate the implant surfaces [43]. The histological analysis showed osseointegration of the previously contaminated part of the implant with all the treatments. However, the amount of bone to implant contact was significantly lower than the non-contaminated part of the implants [44]. In monkeys, CA mixed with saline was applied on the implant surface using gauze 5 times and rinsed. This was followed by a 2 min application of supersaturated CA and then rinsing 20 times with saline. Then, the defects were grafted with autogenous bone graft and covered with ePTFE membranes. This treatment produced approximately 80% bone fill and 43% re-osseointegration at 6 months. When they compared the previously described therapy using AP plus citric acid, CA alone was equally effective [30]. In rhesus monkeys, BMP-2 was used to regenerate the bone after the implant had been decontaminated with CA. There was 40% re-osseointegration 4 months after-surgery [45].

The toxicity of CA has been evaluated in vitro. Apparently CA at 4% to 10% concentrations did not yield cytotoxicity on human osteoblastic cells. However, significant decrease in cell proliferation was reported. Normal proliferation rates were restored approximately 3 days after treatment for the 4% concentration [46]. However, most of the studies reviewed here have reported the use of CA pH1 with a concentration of 40%. An in vitro study showed that CA suppressed the attachment and spreading of fibroblasts on culture plates and Type I collagen. In addition, it was confirmed that the toxic effect of media containing citric acid was due to their acidity rather than the citrate content [47]. The CA application therefore must be limited to the implant surface avoiding the spread of it to the bone and marrow spaces making clinical application of this material difficult.

#### 3.2.2. Chlorhexidine (CHX)

Chlorhexidine gluconate is the most important antiseptic used in periodontics [48]. Its use has been advocated for the treatment of peri-implant conditions as well. Its main indication is for reducing bacterial counts before or after surgical procedures or for the treatment of the periodontal pocket as a local antimicrobial due to its bactericidal properties. Multiple studies have been published about its use to decontaminate the implant surfaces affected by peri-implantitis. An in vitro study demonstrated that rubbing machined, plasma sprayed, and HA coated surfaces for 1 min with cotton pellet soaked with 0.12% CHX reduced the amount of *Porphyromonas gingivalis *(Pg) endotoxin on machined surfaces up to 92.9%, on titanium plasma sprayed surfaces to 62.9% and HA coated surface to 62.8% [40]. 

In vivo, CHX has also shown to contribute to re-osseointegration in dogs. In induced peri-implantitis lesions, after flap elevation the bony lesions were debrided and the implant surfaces cleaned with curettes and rinsed with CHX 0.12%. GTR membranes were adapted and covered with the flaps. The animals were medicated with metronidazole. Significant bone fill from 60 to 80% was obtained and re-osseointegration ranged from 2 to 19.7% [49]. In another study, the implant surface was debrided and subsequently rubbed with CHX soaked gauzed and rinsed with saline approximately 20 times. Implants were randomized to receive autogenous bone grafts and platelet enriched fibrin glue or just CHX. The combined treatment yielded 50.1% re-osseointegration while the CHX group 6.5% [50]. In monkeys, 0.1% CHX applied with a gauze for 5 minutes, followed by rinsing with CHX and saline 20 times, demonstrated 14% re-osseointegration. If the same therapy was combined with autogenous bone graft, there was 22% and with an ePTFE membrane alone 21% re-osseointegration. When combined, CHX, bone graft and membrane yielded 45% [51]. In another study in monkeys, when the contaminated implants were cleaned for 5 minutes with a gauze soaked with CHX and then rinsed 20 times with 0.1% CHX solution and saline alternately, and the defects grafted with autogenous bone and covered with ePTFE membranes, approximately 90% bone fill were obtained and 40% re-osseointegration at 6 months. In this study, the monkeys were also medicated with metronidazole and ampicillin for 12 days [30]. In humans, debridement of the bone defect around the implant and rinsing the exposed contaminated implant surface with 0.1% or 0.2% CHX followed by GTR with non-resorbable membranes has shown to decrease probing depth up to 3 mm and increase bone level by 1.5 mm–3.6 mm [52, 53]. In a randomized clinical trial to evaluate CHX for decrease of total anaerobic bacterial load and putative periodontal pathogens, 48 implants with peri-implantitis were debrided surgically and cleaned with saline soaked gauze and then irrigated with a solution of CHX 0.12% plus 0.5% cetylpyridinium for 1 minute and then rinsed with saline. The control group (31 implants) was irrigated with a placebo solution. The 12-month follow-up showed no statistically significant differences between groups in bacterial counts or clinical markers like plaque, bleeding on probing (BOP), or suppuration [54].

The cell toxicity of CHX on human bone cells has been evaluated. Cellular toxicity seems to be influenced by concentration and exposure time. SEM analysis confirmed absence of osteoblast phenotypic alterations after exposure to 0.2% CHX for 1 minute and CHX 1% for 30 seconds [55]. However, most of the studies discussed in this review have used CHX for longer periods of time than 30 seconds or 1 minute. There is another in vitro study that showed that CHX affected osteoblasts viability in a dose- and time-dependent manners. It induced apoptotic and autophagic/necrotic cell deaths and involved disturbance of mitochondrial function, intracellular Ca^2+^ increase, and oxidative stress [56]. CHX also has shown to inhibit cell proliferation and collagen synthesis [57].

#### 3.2.3. Ethylene Diamine Tetraacetic Acid (EDTA)

EDTA is used in dentistry mainly for its chelating properties. In periodontics it is used to remove the smear layer before applying biomimetic materials for regeneration. EDTA has been also used in implant dentistry. In a randomized clinical trial to evaluate titanium granules for bone grafting in peri-implantitis defects, the surfaces were debrided with titanium curettes and decontaminated with EDTA 24% for 2 min and then rinsed with saline. Patients were medicated with amoxicillin and metronidazole for 10 days. 32 implants were evaluated. There were no statistically significant differences in probing depth (PD) and BOP between groups at 12 months. The EDTA group (control) demonstrated a decrease of PD of 2.6 mm [58]. The main advantage of EDTA is its neutral pH.

#### 3.2.4. Hydrogen Peroxide: (HP)

In vitro, rubbing a cotton pellet soaked with 3% HP for 1 minute was shown to significantly decrease the amount of E. Coli LPS (LPS count 108 min/mm^2^) from titanium alloy grit blasted and HA coated strips compared to untreated controls (LPS count 197 min/mm^2^). However, HP was the least effective when compared to citric acid, plastic sonic scaler tips and air powder abrasive [39]. In another in vitro study designed to evaluate the ability of HP to eliminate *Candida albicans*, *Streptococcus sanguinis*, or *Staphylococcus epidermidis* from titanium specimens, HP was solely effective against *C. albicans* [59]. 3% HP was capable of inactivating attached bacterial cells from human biofilms after immersion in HP for 1 min [41]. 10% HP has also shown to inactivate a human biofilm created in the lab and to eliminate 99.9% of the bacteria attached to the implant surface [60]. Swabbing the implant surface 10% HP for 1 minute has also been shown in animals to decontaminate the implant surface and to allow re-osseointegration to previously contaminated surface in dogs [44].

#### 3.2.5. Saline and Saline Soaked Cotton Pellet

Human studies have shown that combining implant surface cleaning with mechanical methods like curettes and saline soaked cotton pellets contributes to obtaining clinically stable results up to 24 months [18, 20]. An anti-infective therapy including surgical debridement of the implant surfaces with titanium covered curettes or carbon fiber curettes followed by rubbing the implant surface with gauzed soaked in sterile saline and rinsing with saline and with post-operative prescription of amoxicillin and metronidazole for 7 days showed that 88% of the patients and 92% of the implants can prevent the progression of the disease for 12 months [61]. The use of saline soaked cotton pellets to treat induced peri-implantitis in dogs in combination with systemic metronidazole and amoxicillin for 17 days resulted in re-osseointegration of SLA surfaced implants and smooth surfaced implants with significantly more osseointegration for SLA implants [62, 63].

#### 3.2.6. Tetracycline (T)

Tetracycline is a bacteriostatic antibiotic that inhibits protein synthesis. Tetracycline solution has been shown in dogs to allow re-osseointegration. In a study of induced bone defects around implants cleaned with T, there was 1.77 mm of re-osseointegration at 4 months. If DFDBA was added, 2.37 mm of re-osseointegration was obtained [64]. Case reports in humans have shown that a 50 mg/mL of T applied for 5 minutes after implantoplasty or AP and followed by autogenous bone graft or xenograft and membrane resulted in arrest of the disease and radiographic bone fill of the peri-implant defects [9, 65, 66]. 

### 3.3. Lasers

#### 3.3.1. Erbium-Doped: Yttrium, Aluminum Garnet (Er:YAG) Laser

One study found that the irradiation produced by these lasers is poorly absorbed by titanium due to the specific wavelengths; thus, it does not significantly increase the implant temperature [67]. An in vitro study has suggested that their wavelength does not alter the roughness and morphology of smooth and rough surfaced implants, except for minor damage caused by the contact of the tip [29]. Also an in vitro experiment with SLA intraorally contaminated discs treated with Er:YAG and compared to plastic curettes and an ultrasonic system showed that it can effectively remove supragingival early biofilm but fails to restore the biocompatibility of the surface [67].

An in vivo study in dogs demonstrated that Er:YAG laser micoexplosions can remove layers of titanium dioxide from contaminated rough implant surfaces. According to the investigators, the use of water irrigation was able to prevent overheating of the implant protecting the surrounding bone [68]. However, an in vitro study with the Er:YAG found that SLA titanium discs showed alterations after 10 sec of irradiation at 300 mJ/10 Hz characterized by melting down of peaks. On polished titanium surfaces cracks formed with 500 mJ/10 Hz. These alterations of the surface may be associated with a thermal effect due to minimal thermal relaxation between the pulses [69].

In dogs, surface decontamination was performed with an Er:YAG using 62 mJ/20 Hz for approximately 1.5 minutes. The implants were submerged for 6 months after the decontamination. The new bone to implant contact in the defect area was 69.7% compared to 39.4% obtained with plastic curettes used in the control group. The authors stated that with the use of the laser the surface decontamination and granulation tissue removal was achieved without macroscopic visible damage of the surface [70]. However it should be noted that this was a clinical observation and the surface alteration was not measured in any way.

In humans, the use of the Er:YAG laser showed no significant differences in clinical parameters improvement like BOP, PD reduction of CAL, or bone fill when compared with saline soaked cotton pellets at 12 and 24 months of follow-up [19, 20].

#### 3.3.2. Continuous CO_2_ Laser

Under dry conditions a continuous CO_2_ laser has been shown to burn the contaminants but not to remove them [28]. Continuous CO_2_ Lasers used under wet conditions appear to be more successful than dry CO_2_ laser but still fail to remove all the contaminants and to restore the implant surface composition [28]. The use of continuous CO_2_ laser and HP to treat induced peri-implantitis in dogs did not show advantages when compared to saline soaked cotton pellets in terms of the amount of re-osseointegration [63]. Again in dogs, when this laser was compared to AP alone there were no significant differences in re-osseointegration (0.64 mm and 0.58 mm resp.) [31].

Little or no data is available on the other types of lasers for treating peri-implantitis.

#### 3.3.3. Photodynamic Therapy (PDT)

This approach has also been termed light activated disinfection (LAD) and photodynamic activated chemotherapy (PACT). Defined as “light induced inactivation of cells, microorganisms or molecules” [71]. In dentistry, this technique is based on the application of photosensitive dyes activated by a light with a specific wavelength to kill bacteria [72]. It includes three basic elements: visible harmless light, nontoxic photosensitizer, and oxygen. The oxygen is transformed into ions and radicals that are highly reactive and kill the microorganisms [73]. The main photosensitizers found in the literature are hematoporphyrin derivatives (620–650 nm), phenothiazine, like toluidine blue and methylene blue (620–700 nm), cyanine (600–805 nm), phytotherapic agents (550–700 nm), and hytalocyanines (660–700 nm) [71].

In vitro, PDT using methylene blue and GaAIAs low-level diode laser at a wave length of 660 nm when applied for 3 or 5 minutes has shown to significantly decrease bacteria from implants contaminated with saliva of patients with peri-implantitis [74]. In vivo, toulidine blue and GaAIAs low-level diode laser at a wave length of 685 nm for 80 seconds has shown significant reduction and in some cases elimination of pathogenic bacteria associated in peri-implantitis in dogs after surgical treatment [75]. In another study in dogs by the same group, PDT was used with the same protocol. In this study, guided bone regeneration membranes were used after PDT. The results after 5 months showed that there was bone fill up to 48.28% and re-osseointegration up to 25.25% [76]. Evaluation in humans of applying toluidine blue O for 1 minute and then irradiation with a diode soft laser with a wavelength of 690 nm for 1 minute was shown to decrease by 92% of the vital counts of *Porphyromonas gingivalis* (Pg), *Prevotella intermedia* (Pi), and *Aggregatibacter actinomycetemcomitans* (Aa). However, the complete elimination of the bacteria immediately after the procedure was not demonstrated [77]. A clinical study using a similar protocol to treat 40 patients with mechanical debridement with hand, ultrasonic or piezoelectric scalers, and PDT compared to 40 controls without PDT demonstrated that at 4 months there were no significant differences in clinical parameters like PD reduction, BOP, and clinical attachment levels (CAL) between groups [73].

Some factors can influence the PDT effectiveness of surface decontamination including light absorption by the bacteria, wavelength of the laser, time of laser exposure, area to be stained, and the organic matrix of the biofilm [72]. One negative aspect is that currently the dyes do not differentiate between bacteria and host cells; therefore, this could adversely affect the surrounding tissues [25].

One possible advantage of PDT over conventional antibiotic therapy is that this is a topical treatment where only the affected sites requiring antimicrobial treatment receive the dye and illumination limiting the adverse effect seen with systemic antibiotics. Also, there is no evidence of resistance development in the target bacteria after PDT [71].

## 4. Discussion

As more dental implants are placed and remain in function for longer periods the prevalence of peri-implant diseases increases. From this overview of the available literature, it can be said that no reliable and valid therapy can be made based on the published articles available and that the accuracy of the data varies. This agrees with the results of network meta-analysis [6] and systematic reviews [78, 79]. Most of the human studies published are cases series with follow-up periods ranging from 6 months to 24 months making it difficult to determine the stability of the newly formed tissues over time. In the present review it was found that most of the studies do not report rates of implant failures but other surrogate measurements like probing depths or clinical attachment levels. Therefore it is difficult to determine what approach will improve implant survival. This is in agreement with data reported by Faggion Jr. [80, 81].

It can also be stated that presently reattachment of bone to previously diseased implant surfaces is at best unpredictable. Histologic proof of re-osseointegration to previously contaminated implant surfaces in humans was not found. At present a combination of physical and chemical approaches possibly with appropriate laser therapy may prove to provide more predictable results. It should be noted that the profession is early in its understanding of these diseases and their treatment. 

It can be stated with some assurance that physical alteration (smoothing) of the implant surface using metallic instruments has been demonstrated to slow or halt the progression of bone loss in humans as well as animals. While this application is certainly useful, the drawbacks include soft tissue retraction and esthetic compromises. From this review it can be argued that further investigation of optimal ways to treat implants affected by peri-implantitis and peri-implant mucositis as well as the prevention of these problems is warranted.

## 5. Conclusions

Complete elimination of the biofilms needed for reattaching bone to previously contaminated implant surfaces is difficult to achieve. All therapies induce changes of the implant surface chemical and physical properties. However, partial re-osseointegration after detoxification has been reported in animals. Combination protocols for surgical treatment of peri-implantitis in humans have shown some positive results, but long-term evaluation to establish the validity and reliability of the techniques has yet to be determined.
